# Indications for Manipulative Surgery
*Part of an address delivered to the Bristol Medico-Chirurgical Society at the University, on April 11th, 1928.


**Published:** 1928

**Authors:** A. G. Timbrell Fisher

**Affiliations:** Surgeon, with charge of Out-patients, Seamen's Hospital, Greenwich, etc.


The Bristol
Medico-Chirurgical Journal

"*Scire est nescire, nisi id me
Scire alius sciret.*"

AUTUMN, 1928.

### INDICATIONS FOR MANIPULATIVE SURGERY.*

BY

A. G. Timbrell Fisher, M.C., M.B., B.Ch., F.R.C.S.,

*Surgeon, with charge of Out-patients, Seamen's Hospital, Greenwich, etc.*

[After an introduction in which the speaker gave his reasons for believing that manipulative treatment could and should be practised by medical men, particularly by surgeons who had acquired the necessary skill by practice, he went on to give some account of the principles indicating the advisability of using those plans of treatment.]

The types of cases in which manipulation is indicated fall into five principal groups: (1) Cases with adhesions; (2) functional or hysterical cases; (3) unreduced dislocations and subluxations; (4) miscellaneous; (5) combined cases.

* Part of an address delivered to the Bristol Medico-Chirurgical Society at the University, on April 11th, 1928.

### Cases with Adhesions.

Adhesions are bands of scar tissue due to organisation of inflammatory products which give rise to pain, limitation of movement, tenderness, weakness, and recurrent effusion. The most characteristic of these is a certain amount of limitation of movement. They occur frequently after injuries such as sprains, dislocations or fractures, and may be synovial, capsular or extra-capsular. They are very common also after diseases of joints, whether synovitis or arthritis. They are not always associated with joints, but may occur in connection with muscles, tendons and their sheaths, and connective-tissue planes. It cannot be too frequently emphasised that these adhesions are often, indeed usually, preventable by more enlightened treatment of injuries and diseases of joints and of fractures, *i.e.* by instituting early movement.

Examples of post-traumatic adhesions spring at once to mind—the stiffness of the shoulder following dislocation or fracture near the joint, the crippling stiffness of the wrist and fingers following a neglected Colles fracture, stiffness of the ankle and mid-tarsal joint after Pott's fracture, very many cases of flat foot with limitation of inversion, the persistent limitation of full flexion of the knee seen after many cases of sprain.

Why do we still see such large numbers of joints crippled by adhesions ? It is largely due to the persistence of irrational and unphysiological methods of treating injuries and diseases of joints or of the locomotor apparatus generally.

The general treatment, with a view to the prevention of adhesions resulting from synovitis following a sprain or contusion of a joint, can be summed up in a few words, viz., " Rest combined with early movement through complete range." At first sight this maxim may seem contradictory, but this in reality is not the case. Rest from weight-bearing and excessive use permit healing to proceed; but early movements, at first of but short duration, prevent the formation of adhesions. The principle of early movement, admirable though it be, is not sufficient, for unless we aim at early movement *through its full range* adhesions may yet develop and give rise to much subsequent trouble.

Let us attempt to illustrate the principle of the prevention of adhesions by a concrete example, viz. the treatment of a case of synovitis of the knee following a sprain of the internal lateral ligament.

Rest in bed or on a couch is enjoined at first. The next step consists in applying a firm compression bandage over a thick layer of wool, which should completely surround the joint and extend for some little way upwards into the thigh and downwards into the leg, leaving a small fringe of wool above and below uncovered by the bandage.

No splint should ever be used, but the joint is slightly flexed over a pillow, for experience has shown this to be the position of greatest ease. Rest from weight-bearing must be enjoined as long as the synovial effusion persists. From the very first gentle massage (effleurage), electrical stimulation of the quadriceps and movements may be instituted, for these, added to the elastic compression, play an important part in the absorption of the intra-articular effusion and prevent muscular wasting. Each day the movements are increased. In about a week in marked cases, or considerably less in minor cases, effusion will have subsided, and it should be our aim to have obtained full movements by this time.

There is now no objection to weight-bearing, provided strain upon the recently damaged ligament be eliminated, and this can usually be ensured by the simple device of reinforcing the inner sides of the sole and heel by a wedge-shaped piece of leather $\frac{1}{4}$ in. in thickness. For a short period, and especially if there is any tendency to swelling after exercise, a compression bandage should be worn. This, however, should be dispensed with as soon as possible as it tends to interfere with the tone of the thigh muscles. As this swelling is often due to loss of tone of the thigh muscles, every effort should be made to remedy this by re-education.

Then, as examples of stiffness due to disease, we have the crippling disabilities following the so-called "rheumatoid arthritis," when the acuter symptoms have subsided. These cases are literally "crying out" for manipulation, and the risk of a flare-up is negligible if suitable cases are chosen and suitable technique employed. In many of these, of course, the ankylosis is of so firm a nature that manipulation is out of the question, although other surgical measures are full of promise. There is, however, a considerable percentage in which the stiffness is slight or moderate, but sufficient to cause considerable pain and disability. In many instances, by carefully restoring the normal range of movement we break down or loosen the adhesions and scar tissue that are the cause of the greater part of this pain and disability. The results of manipulation in suitable cases of this kind are remarkably good, especially if by suitable after-treatment the wasted muscles are made to recover their tone. I have performed manipulation on many hundreds of such cases, and have been greatly impressed by the possibilities of this method. Many of my patients have had every conceivable form of medical treatment, and this is, of course, logical during the more acute stages; but surely it is scarcely logical to attempt to cure a joint stiffened by scar tissue by vaccines or drugs. The best results accrue in the cases in which the arthritis is or has been of the synovial or capsular type and the actual articular surfaces but little involved.

Adhesions may occur, not only in connection with joints but in muscles or in intermuscular planes, in connection with tendons and their sheaths, fasciæ, aponeuroses and ligaments. Local areas of scar tissue may occur after injuries in connection with these structures which may often be benefited greatly by manipulation. A good example of this lies in adhesions in the lumbar region of the back following an injury to the erector spinæ.

As another example the interesting condition known as "tennis elbow" may be chosen for discussion. That some cases of long standing that have defied every other form of treatment can be cured by manipulation is beyond doubt. The condition is usually attributed to the back hand stroke in tennis or racquets, but it occurs in persons who do not play these games. It is common, for instance, in hammer-men and others who have to grip a thick handle tightly, and some hold that in tennis players it is due to playing with too thick a handle. Clinically there is pain and tenderness in the region of the external condyle of the humerus near the tendinous common origin of the extensor group, or over the posterior aspect of the radio-humeral joint, or both may be combined. There is weakness of grip, so that articles are dropped suddenly. If the fingers are flexed into the palm and the wrist then flexed, there is pain at the site first mentioned. According to Osgood, the symptoms are in many cases due to inflammation of a bursa in this region. I have never, personally, seen any evidence of such a bursa, but think that most cases are due to a partial rupture of tendinous fibres with formation of minute adhesions, and that the cures following manipulation are due to rupture of these adhesions. In some cases periostitis without adhesions seems to be the most prominent feature, and in these manipulation is scarcely indicated.

[The lecturer then pointed out the dangers of manipulation in certain cases, particularly in tuberculous disease and in myositis ossificans.]

*Myositis Ossificans.* — As is well known, this condition is apt to follow an injury of, or in the vicinity of, a joint, particularly the elbow-joint in children, and there is a formation of new bone in muscle, ligament, tendon or other connective-tissue structures. The condition is probably due to the liberation of osteoblasts from beneath the torn periosteum, and it is characteristic that massage, passive movements and manipulation cause increased pain, swelling and muscular spasm, and appear to increase the rate of formation of new bone. There is no objection, however, if the joint has become fixed in a bad position, to manipulating it gently under anæsthesia into that position which experience has shown to be the best should ankylosis unfortunately occur.

### FUNCTIONAL OR HYSTERICAL CASES.

It is in these cases that the most dramatic results are obtained by manipulation. Such cases not unnaturally attract a great deal of public attention; and when, as occasionally happens, the patient has been under the care of, not one, but many eminent surgeons, and the so-called miracle has been performed by a bone-setter, the medical men in question not infrequently come in for some severe criticism.

The diagnosis of a functional condition is often extremely difficult, and depends to a great extent upon that indefinable asset intuition. This is a large and important subject, to which it is impossible to do justice in a short paper, and attention is merely drawn to a few special points which are sometimes overlooked.

1. An injury associated with marked mental shock, such as a railway or motor-car accident in which a joint is injured, is particularly liable to give rise to a neurosis in an individual of a nervous temperament. There is no surgeon of great experience who has not, at some time, been puzzled or misled by a functional condition of the joint.

2. A purely functional disturbance may occur from the first, or more frequently the condition is superadded upon some such basis as a slight degree of chronic synovitis or arthritis. The unwary or inexperienced may overlook the functional element, and consider that the symptoms and signs betoken a much more marked degree of arthritis than is actually present.

3. Quite apart from the question of injury, it must be remembered that a functional element is very frequently quite a marked feature in chronic arthritis in both sexes. These are the cases of "rheumatoid arthritis" of many years' standing which are cured by "faith-healers."

4. In an early functional case, if spasm be abolished by anæsthesia, full movement can be obtained readily. If, however, the condition is of long standing, actual adaptive shortening of ligaments and periarticular structures occurs. This is, of course, more liable to occur if the condition has been superimposed upon a slight degree of chronic synovitis or arthritis.

5. Even in primarily functional cases swelling of the joint may occur, probably from the general loss of tone of the thigh muscles and impaired absorption of synovial fluid. It may then be a moot point whether the functional condition is primary or secondary.

6. Manipulation is by far the most effective therapeutic measure in these functional cases owing to the powerful suggestion of the anæsthetic. Treatment by suggestion should, however, precede and immediately follow the anæsthetic, and be carried on for some weeks or months subsequently.

7. The after-treatment is often of more importance than the manipulation itself, and not only the surgeon, but everyone with whom the patient comes in contact, must endeavour to convince the patient that he or she is now cured.

8. The prognosis in these cases depends upon the time that the condition has been present. In cases of long standing the prognosis is less hopeful. Even more, perhaps, depends upon the personality of the surgeon who performs the manipulation.

The type of case most frequently encountered is the chronic back, with which we are all probably familiar. There is usually a history of some injury, of moderate severity, such as a fall, thereafter there is a complaint of a dull ache in the back after any exercise, and the patient gradually becomes a semi-invalid, X-ray examination is negative, and other causes of pain in the back have been excluded. There is usually pain on stooping and recovering, and the back muscles are flabby. Very often these patients have either been told that there is nothing really wrong with the back or, on the other hand, have been led to believe that serious trouble is present. Not infrequently they have worn spinal jackets and supports over prolonged periods without relief. In this type of case the results of manipulation, followed by re-education of the spinal muscles, are often almost miraculous.

### DISLOCATIONS AND SUBLUXATIONS.

This is another large group of cases in which manipulation is of the greatest value. The treatment of the ordinary dislocations by the classical methods is well known and need not detain us. A few words are necessary, however, concerning dislocations, or, to speak more accurately, "fracture-dislocations" of the semilunar cartilages. It has always been a profound mystery to me why this condition, so absorbingly interesting and so frequently encountered in practice, has been so neglected and left largely to bone-setters. It is extremely probable that a very large percentage of the patients of a well-known bone-setter recently retired, consisted of unrecognised cases of unreduced fracture - displacement of the semilunar cartilage. No little part of his reputation was due to his undoubted skill in these manipulations. It is regrettable that many of these patients had sought help from our profession in vain.

There is evidence that manipulation is not only of value in recent cases of fracture-dislocation of a semilunar cartilage, but also in some recurrent cases. A fracture-dislocation of a semilunar cartilage must be reduced immediately, and reduction has not occurred until the patient can fully and painlessly extend the knee. If extension is limited by only a few degrees, or if by an effort one can get almost full extension, but this is still painful, the cartilage is probably still unreduced. With appropriate treatment there should be no recurrence.

In the recent cases we must bear in mind that the displaced fragment, owing to its elasticity, has a pronounced tendency to spring back into position, provided the obstruction presented by the femoral condyle can be eliminated. I advise every one of my listeners who has not done so to take a femur and tibia, articulate them, and settle for himself what are the manipulative movements that will most readily cause the displaced fragment to retrace its step. He will acquire a more accurate conception than by a perusal of the usually faulty text-book descriptions. The latter often advise a long period of immobility after reduction, and some even say that the limb should be put in plaster for a period. As a matter of fact, provided rotatory movements are avoided, the patient can start gentle exercises as soon as the acuter symptoms have subsided and effusion almost gone. Straightforward flexion and extension, combined with weight bearing, probably assist repair. Prolonged immobility leads to marked and sometimes irremediable wasting and stiffness. Permanent stiffness of a limb is not a cure for a torn cartilage.

In some chronic cases apparent cure occurs, but in some the benefit is of a temporary nature. This fact depends upon the physiology of repair in the semilunar cartilages. Repair is at best sluggish, and when the fractured surfaces have become smooth and endothelialised, as in cases of long standing, it is probable that actual repair occurs but rarely. How, then, can we explain the relief or even apparent cure that manipulation may bring about in these chronic cases?

A fact of fundamental importance in this connexion is that the commonest lesion of a semilunar cartilage is a definite tear which usually takes the form of a complete longitudinal tear or "bucket-handle" type of lesion, in other words it is a "fracture-dislocation." The characteristic symptoms of the semilunar derangement are due to the behaviour of the fractured portion of cartilage. It is most important to remember that in these recurrent cases it is quite possible for the inner torn portion to occupy a certain position without giving rise to any " locking " or limitation of movement or, indeed, to any physical or clinical symptoms whatsoever. This position is the narrow space in the intercondylar region between the crucial ligament and the femoral condyle.

During rotation of the tibia upon the femur rotation takes place in the arc of a circle, the centre of which is at the tibial attachments of the crucial ligaments. It follows that during any sudden rotatory movement the movement in the interior of the joint near the centre of the circle (near the crucial ligaments) is necessarily less than at the periphery. It follows, also, that in a given case of chronic " bucket-handle " lesion of a semilunar cartilage, in which the surfaces have become smooth and rounded and actual repair probably impossible, the displaced fragment might be less likely to give rise to symptoms in the position aforesaid than if actually restored to its original position.

I have performed successful manipulations upon many of these recurrent cases of semilunar lesion with the avowed intention of driving the fractured portion into the interior of the joint. In my opinion a successful result depends upon converting an incomplete longitudinal tear into the complete variety, and the manipulative technique differs appreciably from that necessary for recent cases.

It may be argued that at operation in these recurrent cases the displaced fragment is usually found in the interior of the joint, where it obviously gave rise to symptoms. However, in most of these cases the tear is not absolutely complete, but is such that a certain degree of tension is present, sufficient to cause recurrent displacements.

To sum up, the commonest lesion of a semilunar cartilage is a definite tear, and most commonly the complete longitudinal tear or "bucket-handle" type. At the original accident the inner torn portion is usually dislocated into the interior of the joint, where, being fixed at first between the femoral and tibial condyles, it gives rise to a mechanical block to extension.

By manipulation two things may happen: (1) In more recent cases the displaced portion may retrace its steps and, provided rotary movements are avoided for some time, repair may take place—in more chronic cases a trial may be given to the same method; and (2) if it fails the displaced portion may be forced still farther into the interior of the joint, where, in many cases, it may give rise to no further trouble for considerable periods, although recurrences often occur.

Manipulation in recurrent displacements of the semilunar cartilages, although well worth trying, is not infallible, particularly in professional footballers and others whose work is strenuous. It is well worthy of trial in persons whose work or play is not of a strenuous nature. The results of operative removal of the torn cartilages are so satisfactory in experienced hands that this should be usually advised when there is a history of several recurrences. The patient should be up and walking fairly well ten to fourteen days after the operation, and although one occasionally hears a contrary opinion expressed, experience teaches that removal of the cartilage does not prevent participation in the more strenuous forms of work or sport.

Within the short space at my disposal it has obviously been only possible to touch briefly upon a few aspects of treatment by manipulation. I trust, however, that sufficient has been said to indicate the very great possibilities of affording relief or of curing disability that lie in this neglected field, to all who believe that Dogma is the enemy of Progress.

